# Case report: Autonomic and endocrine response in the process of brain death in a child with hypoxic-ischemic brain injury

**DOI:** 10.3389/fped.2022.954651

**Published:** 2022-07-22

**Authors:** Kenichiro Hayashi, Kaname Uchida, Hidehito Ota, Hiroyuki Tanaka, Mieko Maezawa, Hikoro Matsui

**Affiliations:** Department of Pediatrics, The University of Tokyo Hospital, Tokyo, Japan

**Keywords:** autonomic nervous system, brain death, cardiac arrest, child, heart rate variability, hypoxic-ischemic brain injury

## Abstract

**Background:**

The causes of brain death include cerebral herniation and brainstem ischemia. Neuroendocrine failure or a series of autonomic nervous system disorders are clinically recognized in the transition to brain death among patients with critical brain injuries. An accurate evaluation of these physiologic instabilities and biomarkers is essential to assess the severity and prognosis of pediatric brain injury as well as to initiate supportive care. This case report presents a detailed evaluation of the autonomic nervous system and endocrine function during the transition to brain death in infantile hypoxic-ischemic brain injury by analyzing the heart rate variability and endocrine status.

**Case Presentation:**

A 1-year-old previously healthy boy went into cardiac arrest after choking on a toy at home. Although spontaneous circulation returned 60 min after cardiopulmonary resuscitation, no cerebral activity or brainstem reflexes were observed after 18 hospital days. The heart rate variability was assessed by analyzing the generic electrocardiogram data. Rapid spikes or drops in the total power of the heart rate variability, accompanied by a cortisol surge, as well as an alternating surge of high- and low-frequency domain variables were detected in the process of brain death.

**Conclusion:**

The heart rate variability assessment combined with endocrine provides a better understanding of the clinical course of patients undergoing brain death. It accurately detects the loss of brainstem function, which allows physicians to provide the appropriate supportive care.

## Introduction

Severe brain injury with cerebral herniation and brainstem ischemia can lead to brain death, which is characterized as a deep comatose state, bilateral pupil dilation and fixation, loss of brainstem reflexes, flattening of the electroencephalogram, and apnea (1). In the transition to brain death, neuroendocrine failure or autonomic nervous system (ANS) disorders, including Cushing’s triad (bradycardia, hypertension, and irregular breathing), autonomic storm (hypertension and tachycardia), and subsequent hypotension, can be typically observed. These physiological phenomena vary depending on the pattern and severity of ANS injury, and not all of the changes are observed in each patient declared brain dead (2). In the field of transplant medicine, a precise evaluation of the process rather than the outcome of ANS dysfunction is vital, therefore, in understanding the transition period to brain death and in initiating organ-protective care.

Brain plasticity, which indicates the capacity of the brain to recover its functions after an injury, varies among neonates, infants, younger and older children. Therefore, it is of paramount importance to evaluate in detail the clinical course of each individual patient after brain injury. The physiological alterations, closely monitored through a detailed assessment of ANS and endocrine function, have the potential to predict the clinical course of severe brain injury, particularly among pediatric patients. Specifically, ANS function serves as a point-of-care testing for the severity and prognosis of pediatric brain injuries (3–5). Further monitoring the alterations in ANS function over time can reveal the changes in the level of brain damage, because the two components of ANS, the sympathetic nervous system and the parasympathetic nervous system (PNS), form a network with several brain regions, including the cortex, limbic system, midbrain, and brainstem (6).

Heart rate variability (HRV) is a standard method for assessing ANS function (7). HRV analyses fluctuations of regular heart beats generated through efferent sympathetic and parasympathetic activity from the medulla, integrated with activity occurring in the intrinsic nervous system of the heart (7). HRV can be a sensitive indicator of medullary integrity and is associated with critical conditions of the central nervous system in children, including neonatal hypoxic-ischemic brain injury (HIBI), traumatic brain injury, and brain death (3, 5). Hence, the combined assessment of HRV and neuroendocrine function in HIBI may indicate a process of pediatric brain injury that cannot be detected using clinical findings alone.

This case presents a detailed evaluation of ANS and endocrine function during the transition to brain death in infantile HIBI by analyzing HRV and endocrine status.

## Case presentation

**FIGURE 1:**
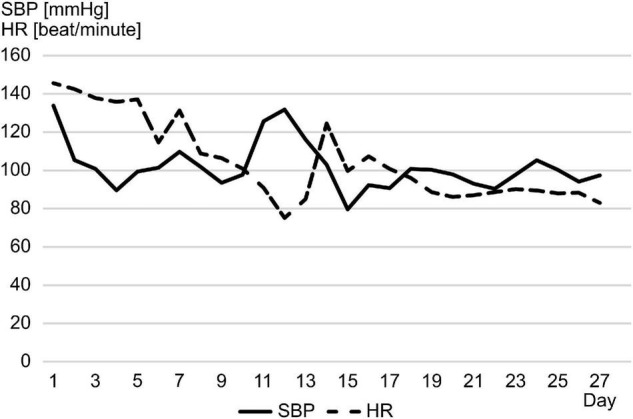
From day 11 to 13, the patient’s systolic blood pressure increased rapidly with bradycardia, which appeared to be a Cushing reflex. HR, heart rate; SBP, systolic blood pressure.

**FIGURE 2:**
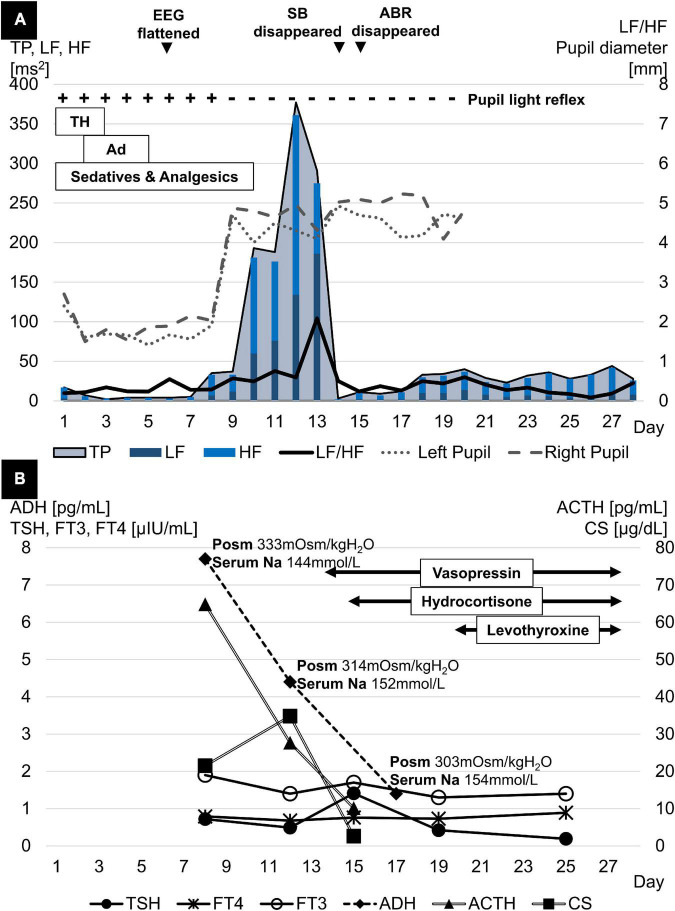
**(A)** TP rose rapidly in parallel with bilateral pupil dilation and fixation, reached a peak a few days later, and then dropped sharply, the timing of which coincided with the disappearance of spontaneous breathing and the full waves of ABR. LF/HF peaked a few days after TP and declined simultaneously with TP. **(B)** ACTH levels declined dramatically in parallel with progressive brain damage. ADH levels also showed a similar trend despite the higher Posm. Meanwhile, FT3 and FT4 levels both fluctuated at low levels with normal TSH levels after initial brain damage. ABR, acoustic brainstem response; ACTH, adrenocorticotropic hormone; Ad, adrenaline; ADH, antidiuretic hormone; EEG, electroencephalogram; FT3, free triiodothyronine; FT4, free thyroxine; HF, high frequency; LF, low frequency; Na, sodium; PLR, pupillary light reflex; Posm, plasma osmolality; SB, spontaneous breathing; TH, therapeutic hypothermia; TP, total power; TSH, thyroid-stimulating hormone.

A 1-year-old previously healthy boy went into cardiac arrest after choking on a 5-cm toy at home. Cardiopulmonary resuscitation was performed by a bystander, and spontaneous circulation returned after 60 min. He was transferred to the pediatric intensive care unit for management of post-cardiac arrest syndrome. Therapeutic hypothermia was induced within the first 48 h to achieve a body temperature of 34.5°C and to protect his central nervous system from secondary injury. He was prescribed mannitol from day 1 to 16 to reduce intracranial pressure (ICP), adrenaline from day 2 to 5 to stabilize hemodynamics, and dexmedetomidine, midazolam, and fentanyl for sedation and analgesia from day 1 to the end of day 10. The patient remained in a deep comatose state with a Glasgow coma scale score of 3 even after the cessation of sedative or analgesic drug administration. The electroencephalogram flattened on day 6, computed tomography findings indicated a low density area and edema throughout the cerebrum on day 7, and bilateral pupil dilatation and fixation were observed on day 9. The Cushing’s triad was observed from days 11 to 13. Spontaneous breathing disappeared and central diabetes insipidus developed on day 14, and all auditory brainstem response waves were undetectable starting from day 15 (Figures 1, 2A). On day 18, two pediatric neurologists declared that the patient fulfilled the criteria for brain death, except for the apnea test. On days 23 and 24, legally mandated tests for determining brain death were conducted, and no cerebral activity or brainstem reflexes were found.

The HRV was assessed by analyzing generic electrocardiogram data, recorded on an exclusive server (Clinical Database Engine; Phillips, Amsterdam, Netherlands), with a sampling frequency of 500 Hz from a bedside monitor (IntelliVue MX800; Phillips, Amsterdam, Netherlands). R-wave peaks were extracted using an automated QRS detection algorithm, and the R-to-R intervals were analyzed to calculate the HRV using the Kubios HRV Standard (ver. 3.5.0) (Biosignal Analysis and Medical Imaging Group, Kuopio, Finland). The fast Fourier transformation was used to retrospectively measure frequency domain variables, particularly the total power and the ratio of low frequency [(LF): 0.04–0.15 Hz] component to high frequency [(HF): 0.15–0.4 Hz] component power, on a 24-h basis. Artifacts in R-to-R intervals were removed using the threshold-based beat correction algorithm of the Kubios software (8). From days 9 to 14, the sharp increase in total power coincided with bilateral pupil dilatation and fixation. Meanwhile, the steep decrease in total power was linked to the disappearance of spontaneous breathing and all auditory brainstem response waves (Figure 2A). Endocrine testing showed a significant decline in pituitary hormone levels in parallel with the progression of brain damage. Cortisol (CS) reached its peak on day 12 and then dropped rapidly. Free triiodothyronine and free thyroxine levels fluctuated at low levels whereas thyroid-stimulating hormone levels remained normal (Figure 2B).

## Discussion

This pediatric HIBI case demonstrated two physiological findings related to brain death. First, the rapid increase and decrease in total power were accompanied by a CS surge. Second, the alternating surge in HF and LF occurred along with an increase in the LF/HF ratio. The first significant finding developed as the secondary injury progressed from the cerebrum to the brainstem over several days. There was a dramatic rise and fall of the total power, reflecting variance in overall ANS activity (7). Since the brainstem is more resistant to anoxic injury than the cerebrum (9), cerebral edema, caused by reperfusion-induced ischemic injury, occurs first in HIBI. This is followed by cerebral herniation through the foramen magnum due to increased ICP and brainstem ischemia (2). Throughout the course of deterioration, ANS is activated in response to the elevated ICP to maintain cerebrovascular homeostasis until the ICP exceeds the threshold at which ANS collapses due to impaired cerebral blood flow (10). In this case, the steep increase and decrease in total power were accompanied by a CS surge. This finding represents a moment of stress on the moribund brainstem following brain edema. In contrast, adrenocorticotrophic hormone, secreted from the anterior pituitary gland, and anti-diuretic hormone, secreted from the posterior pituitary gland, rapidly decreased, most likely because the blood supply to the hypothalamus and the pituitary gland ceased as the cerebral or brainstem injury progressed (11). In addition, both free triiodothyronine and free thyroxine levels fluctuated at low levels whereas thyroid-stimulating hormone levels remained normal, consistent with a phenomenon known as non-thyroidal illness syndrome (low T3-low T4 syndrome) due to severe physiological stress (11).

Another finding was the alternating surge in HF and LF, which was accompanied by the increase in LF/HF. This was observed as the injury spread from the cerebrum to the brainstem. LF/HF reflects sympathetic activity (10) because the HF component is affected by the PNS, while the LF component is influenced by both the sympathetic nervous system and the PNS. This case was unique because the PNS was predominantly activated on day 12, and the sympathetic nervous system was predominantly activated 24 h later on day 13. In previous reports, either the HF or LF predominantly increased as the ICP increased (10, 12, 13). The decreased blood flow secondary to ICP elevation possibly stimulates the PNS, resulting in cerebral vasodilation. This contributes to the maintenance of cerebral blood flow (10). This mechanism leads to further ICP elevation (10) which in turn overdrives the sympathetic nervous system as a biophysical stressor (12).

Some confounding factors may have affected HRV during this patient’s treatment course. Therapeutic hypothermia can increase HRV (14), whereas adrenaline, sedatives, and analgesics can reduce HRV (15, 16). In the present case, neither total power nor LF/HF appeared to change after the cessation of therapeutic hypothermia or adrenaline. Sedatives and analgesics were administered until several days before total power peaked, while total power began to rise before they were discontinued. Therefore, the above factors are unlikely to have affected HRV in this patient and are more likely to have reflected intrinsic ANS function.

The utility of HRV assessment in pediatric intensive care is to visualize the process of brain injury for clinicians and parents, apart from clinical symptoms. Currently, HRV has not been established for bedside vital monitoring (4). However, the analysis could be applied to semi-real-time monitoring for bedside assessment of ANS function in future, since HRV can be analyzed in a shorter time frame of 5 min rather than 24 h (7). If applicable, HRV has the potential to provide additional clinical information on the pathophysiology of brainstem function, which cannot presently be analyzed in real time.

In conclusion, pediatric HIBI is a fatal condition, that presents with rapid increases and decreases in the total power of HRV, accompanied by a CS surge, as well as an alternating surge in HF and LF, accompanied by an increasing LF/HF, during the transition to brain death. These physiological changes in the HRV and the neuroendocrine function allow further understanding of the clinical course, particularly the loss of brainstem function. This finding allows physicians to determine the appropriate supportive care.

## Data availability statement

The raw data supporting the conclusions of this article will be made available by the authors, without undue reservation.

## Ethics statement

The studies involving human participants were reviewed and approved by the IRB Board of The University of Tokyo Hospital [Institutional Review Board No. 2701–(5)]. Written informed consent to participate in this study was provided by the participants’ legal guardian/next of kin.

## Author contributions

KH contributed to the data collection and drafted the initial manuscript. HM supervised the manuscript. KU, HO, HT, and MM contributed to reviewing and editing the manuscript. All authors agreed to be accountable for the content of the work and approved the submitted version.

## Conflict of interest

The authors declare that the research was conducted in the absence of any commercial or financial relationships that could be construed as a potential conflict of interest.

## Publisher’s note

All claims expressed in this article are solely those of the authors and do not necessarily represent those of their affiliated organizations, or those of the publisher, the editors and the reviewers. Any product that may be evaluated in this article, or claim that may be made by its manufacturer, is not guaranteed or endorsed by the publisher.
